# The "Rideal-Walker" Method of Standardising Disinfectants

**Published:** 1907-12-21

**Authors:** 


### Public Health and Hygiene.

### THE "RIDEAL-WALKER" METHOD OF STANDARDISING DISINFECTANTS.

We review a brochure upon this subject in our present issue, and some of our readers may be interested to know briefly what the Rideal-Walker process is.

There is still considerable confusion in the public, and possibly even in the professional, mind as to the exact meaning of the term disinfectant.

A *deodorant* may or may not be a disinfectant, for example; indeed, a substance may have a powerful effect as a deodoriser, and yet may have very little disinfectant power at all. There is a prevalent delusion that bad smells are themselves the cause of disease, and many persons who would never dream of stopping in a place where there is an ill-smelling drain or the like, feel quite secure if the smell be removed or overcome by a deodorant. They forget that the bad smell is but a provision of nature to drive them away from the place where putrefactive and other microbes are multiplying; and that to remove the smell without being sure that the microbes are removed or killed too, is to lull themselves into a sense of security amidst a danger which may have been rather increasing than diminishing.

Even an *antiseptic* need not be a disinfectant, though the terms antiseptic and disinfectant are often used as if they were interchangeable and synonymous. An antiseptic may be a disinfectant, and often is; but there are many antiseptics which are not disinfectants at all. The term antiseptic premises that the substance to which it is applied has the power of inhibiting the growth of micro-organisms. Sugar and salt are two substances used every day in domestic processes for the prevention of putrefaction; as such they are antiseptics in the strict meaning of the term, but they are not disinfectants.

The essential feature of a *disinfectant* is that it possesses the power of killing micro-organisms; and if a synonym is required, *germicide* is the term which is its nearest equivalent.

Mr. Partridge, in his brochure, very rightly points out that there are many desiderata in an ideal disinfectant, namely:—

1. It should possess a high germicidal power. 2. It should be capable of being used in the presence of organic matter without any great diminution of its germicidal power. 3. It should be homogeneous and capable of retaining its homogeneity. 4. It should yield a solution or emulsion in all proportions. 5. It should be innocuous to man and the higher animals. 6. It should be free from corrosive action on the skin and on metals. 7. It should have the power of penetration. 8. It should be reasonable in price when diluted to a working solution. 9. It should be a deodorant.

It is clear that no disinfectant known to us at present is perfect in all these respects; we can only do as well as possible with the means at our disposal. It is also clear that it by no means follows that the disinfectant with the greatest germicidal power is the best to use, for it may not possess penetrating powers, or it may corrode metals, and so on. Nevertheless, the one desideratum that is most essential is germ-killing power, for without this a substance is not a disinfectant at all. It is generally agreed, therefore, that the basis for standardisation of disinfectants must rest largely upon the power of each to kill germs.

Various methods have been employed with this end in view; the best known are the "Garnet," the "Thread," and the "Rideal-Walker." The latter consists essentially in testing the germicidal power of a given disinfectant, and that of a known dilution of carbolic acid, simultaneously, and expressing the microbe-killing power of the former in terms of carbolic acid. In other words, the "Rideal-Walker" process takes carbolic acid to be the standard by which all other disinfectants should be measured. If this be done, it is possible to say that the germicidal power of a given disinfectant is 5, 10, 20, 30, or 40 times that of standard carbolic acid, and so on; the figures obtained being termed the "carbolic acid coefficient" of any particular disinfectant that may be under consideration.

All this seems to be simplicity itself, at first sight; but endless difficulties have arisen for two chief reasons—namely:

1. The germicidal power of disinfectant A. has not always been estimated under precisely the same conditions as that of the standard carbolic acid.

2. The germicidal power of carbolic acid itself is very different in distilled water from that in fluids containing various percentages of organic matter.

The result of the first of these has been that manufacturers have advertised their disinfectants as having this or that carbolic acid coefficient, when really the true coefficients are nothing of the kind. If the Rideal-Walker method is to be adopted, it is essential that it should be followed accurately and in a precisely similar way in all cases. The technique of the method is not difficult, and a full description of it is given in Mr. Partridge's booklet as well as in the original papers by Rideal and Ainslie Walker. The usual organism employed is the bacillus typhosus, but it is not sufficient to take a culture of typhoid bacilli, mix some disinfectant A. with it, see how long it is before all the bacilli are dead, and then say that this is so many hours or minutes quicker than it was when carbolic acid was used on a former occasion. It is essential that the carbolic acid control should be performed at the same time, in the same time, and under precisely similar conditions as the testing of disinfectant A. It is the absence of *simultaneous* contral experiments with carbolic acid which has led to much of the present controversy. The things which must be identical both for the disinfectant that is being tested and for the carbolic acid control are: (1) The time of the experiments; (2) the age and species of the culture used; (3) the nature and reaction of the medium; (4) the temperature of incubation; (5) the temperature of medication; (6) the proportion of culture to disinfectant. If these points are all attended to, then the figures given by manufacturers will be of value, for they will give real carbolic-acid coefficients and not fictitious ones.

The other difficulty is a greater one. The germicidal power of disinfectants varies greatly according to the amount of organic matter present in the fluid in which it is tested, and the variations do not run parallel when different germicides are being tested. The disinfectant power of carbolic acid is very much greater in distilled water than it is in water to which 1 per cent. or 2 per cent. of organic matter has been added. When as much as 15 per cent. of organic matter is present, the germicidal powers of all disinfectants are reduced almost to the same dead level. The question at once arises: if the Rideal-Walker process of estimating the carbolic acid coefficients of different disinfectants is to be adopted as the universal standard, ought it to be carried out with carbolic acid in distilled water, or ought organic matter to be present? If the latter, then what percentage of organic matter ought to be recognised as the standard? Obviously the germicidal action of disinfectants is nearly always required in the presence of some organic matter, but there is as yet no general agreement amongst those concerned as to how much, if any, should be admitted when the carbolic acid coefficients are being estimated. Some dilute with sterilised water; others, as in the Sommerville-Walker modification of the test, use 1 or 2 per cent. of some organic material such as the constituents of urine. It is clear that uniformity is required, and it is equally clear that this uniformity has not yet been attained. Until it is, the medical practitioner can only look on and watch the controversy that is at present raging upon the question, and he may have been glad to have had this brief survey of what the controversy is about.

